# The prediction of ICD therapy in multicenter automatic defibrillator implantation trial (MADIT) II like patients: a retrospective analysis

**Published:** 2008-04-01

**Authors:** Marco Budeus, Nico Reinsch, Heinrich Wieneke, Stefan Sack, Raimund Erbel

**Affiliations:** Department of Cardiology, West-German Heart Centre, University of Duisburg-Essen, Germany

**Keywords:** MADIT II, electrophysiologic study, appropriate ICD therapy

## Abstract

**Objectives:**

MADIT II like patients have not been compared to patients without an electrophysiological study, patients in whom ventricular tachycardia or fibrillation were induced in an electrophysiological study (EPS) and patients without an inducibility in EPS in one study.

**Background:**

The multicenter automatic defibrillator implantation trial (MADIT) II showed a benefit of ICD implantation in patients with ischemic heart disease.

**Methods:**

We performed a retrospective analysis in 93 patients with an ischemic heart disease and an ejection fraction ≤30% who had an ICD implanted with a follow-up at least an 18 months. Patients were divided into 3 groups according to the primary indication for ICD implantation: without EPS (group I), patients in whom ventricular tachycardia or fibrillation were inducible in EPS (group II) or patients without an inducibility in EPS (group III).

**Results:**

During the mean follow-up of 32.9 ± 16.1 months 289 appropriate ICD therapies and 10 deaths occurred. The incidence of appropriate ICD therapies did not differ significantly between the groups (group I 40%, group II 54% and group III 48% of patients). We found in group II a higher risk of appropriate ICD therapies with occurrence of a specific constellation of EPS values. These patients showed a 15-fold risk (P = 0.005) of an appropriate ICD therapy. Furthermore a brain natriuretic peptide value of 265 pg/ml also predicted an appropriate ICD therapy with a 3.5-fold risk (P = 0.017).

**Conclusion:**

In the present retrospective study the results of MADIT II were affirmed in the case of incidence of ventricular arrhythmias in patients with an EF < 30% and coronary heart disease. The prediction of an appropriate ICD therapy with EPS was only achieved in patients with inducibility in the EPS.

## Introduction

The multicenter automatic defibrillator implantation trial (MADIT) II showed a reduction of the risk of all-cause mortality by 31% over an average follow-up of 20 months in patients with an ejection fraction ≤30% and coronary heart disease [1]. The cost-effectiveness of ICDs after the implementation of the MADIT II results into the guidelines was discussed controversial [2-6].

To the best of our knowledge there is no study which compared MADIT II like patients who did not undergo an electrophysical study (EPS), MADIT II like patients in whom ventricular tachycardia or fibrillation were inducible in EPS and MADIT II like patients without an inducibility in EPS in one study. Daubert et al. compared the MADIT II patients with and without inducibility in EPS but they did not compare those patients who underwent an EPS to patients who did not undergo an EPS [7]. It is unclear whether MADIT II patients provided from an EPS or a subgroup of patients could be identified with higher risk for ICD therapy.

Therefore we performed a retrospective analysis of MADIT II like patients with regard to an appropriate ICD therapy. In addition we performed an analysis of demographic data, NYHA classification, brain natriuretic peptide (BNP) and echocardiographic measurements for the prediction of an appropriate ICD therapy.

## Methods

We enrolled 93 patients with coronary heart disease and myocardial infarction who have had a follow-up at least of 18 months between January 2003 and December 2006 in a retrospective study. The myocardial infarction had to be at least one month prior to ICD implantation and the EF had to be ≤30%. The ICD was implanted because of inducibility of ventricular fibrillation (VF) (11 patients) or monomorphic ventricular tachycardia (VT) (18 patients) in EPS (group II), in 27 patients without inducibility in EPS (group III) or a primary indication (37 patients = group I) due to the MADIT II criteria [1] without performing an EPS. The EPS was performed in patients of group II and III because of unclear results (artefacts, triplet, bigeminy, non-sustained VT) in 24h-Holter ECG. Defibrillator systems were manufactured by Biotronik GmbH & Co (Berlin, Germany), Guidant Corp. (St. Paul, MN), Medtronic Inc. (Minneapolis, MN) and St. Jude Medical CRMD (Sylmar, CA) (alphabetical order).

Antitachycardia therapy was standardized providing for VT cycle lengths raging from 450 to 300 ms. The cut-off cycle length of ICD with immediate shock delivery (VF zone) was < 300 ms in all patients, Antibradycardia pacing was programmed with basic drive of 40 bpm/min for single chamber ICD, and 50 bpm/min for dual chamber or CRT ICD's. The study protocol was approved by the institutional review board.

### Study end points

The primary end point of the study was the occurrence of an appropriate ICD therapy. The secondary end point was death.

### Electrophysiological  study and definition of inducibility

In all patients of group II and III an EPS was performed with up to 3 extrastimuli from two different right ventricular sites during sinus rhythm or atrial fibrillation and basic drive pacing (8 beats) at cycle lengths of 500, 430, 370 and 330 ms with a S2 and S3 stimulation as well as at cycle lengths of 500 ms with a S4 stimulation. If it was required S4 stimulation was performed at cycle lengths of 500 ms. The ventricular extrastimulus was twice the diastolic threshold and 2 ms duration. Extrastimuli were decrementally down to the coupling interval no shorter than 200 ms or to the ventricular refractory period. The endpoint of the EPS was the completion of the stimulation protocol or induction of  VF, sustained monomorphic or polymorphic VT. Rapid burst pacing was not used for induction of VF.

A sustained ventricular tachycardia was defined as lasting 30 sec or requiring termination sooner because of a hemodynamic compromise. A monomorphic VT was defined as VT with a uniform beat-to-beat surface QRS morphology. A polymorphic VT had a variable surface QRS morphology, and VF was defined as a rapid, disorganized rhythm without  identifiable QRS complexes.

We analysed retrospectively the data of EPS in order to reach a subdivision of patients with induction of VF, sustained monomorphic or polymorphic VT. First we performed a ROC analysis for the particular S2, S3 and S4 refractory periods of patients without induction of ventricular arrhythmias and the induction interval of patients with inducibility of ventricular arrhythmias. Second we analysed the differences of the particular S2, S3 and S4 refractory periods (∆P) of patients without induction of ventricular arrhythmias and the induction interval of patients with inducibility of ventricular arrhythmias. Therefore we calculated the differences (∆P) between S2 vs. S3, S2 vs. S4 and S3 vs. S4.

We defined an abnormal EPS as following: First extrastimulus below 400 ms and ∆P of ≤20 ms between second (S2) and third (S3) extrastimulus or first extrastimulus above 400 ms and ∆P > 20 ms between second and third extrastimulus or whether a 500 S4 was performed the difference between third and fourth extrastimulus of ≤20 ms in patients with inducibility of ventricular arrhythmias. A definition of an abnormal EPS in patients without inducibility achieved no significant difference in patients with or without ICD therapy with these methods.

### Analysis of ICD therapy

We used the interrogable data of the ICD to analyse appropriate ICD therapy every three months. ICD therapy  for VF or VT was defined as appropriate therapy, and ICD therapy  for atrial fibrillation, supraventricular tachycardia, sinus tachycardia or abnormal sensing was defined as inappropriate therapy.

A VT was characterized with uniform and regular complexes between 170 and 250 beats/min. VF was defined as an irregular rhythm with > 200 beats/min and indistinct electrogram complexes. A polymorphic VT was classified as VT when < 200 beats/min and as VF when the rate was > 200 beats/min [1]. VT was differentiated from supraventricular tachycardias using standard criteria, including a change in electrogram morphology, sudden onset and atrial-ventricular relationship if atrial electrograms were available.

### Brain natriuretic peptide

Patients have had their venous blood samples drawn from a forearm vein for BNP concentration. BNP measurement (Triage Meter Plus®, Biosite GmbH, Willich, Germany) was performed randomly during working hours in all patients 1-3 days before ICD implantation. The detection limit was 5 pg/ml and upper measurement limit was 5000 pg/ml. Samples were analysed by personnel blinded to the patients clinical data. We performed a retrospective analysis of our data to define a margin.

### Echocardiography

The physicians were blinded to the clinical data of the patients. Biplanar left ventricular end-diastolic and end-systolic cavity volumes were calculated using Simpson's rule [8] from paired apical four-chamber and apical long-axis echocardiographic images of a minimum of five cardiac cycles; mean values of each variable were estimated. Biplanar ejection fractions were calculated as end-diastolic volume - end-systolic volume / end-diastolic volume x 100% [9]. Left ventricular systolic and end-diastolic diameter of all patients was measured by M-mode and two-dimensional echocardiography using the Phillips ultrasonic device (3.5 MHz; model Sonos 5500, Philips Medical System, Andover, Massachusetts, USA).

### Statistics

All data are presented as mean values ± standard deviation for continuous variables and as percentages for categorical variables. Discrepancies in proportions were evaluated for statistical significance using the chi-square or Fisher's exact test. Student's t-test was used comparing continuous variables except of BNP value, as its distribution was skewed. Here, the Mann-Whitney U test was employed. All statistical tests were two-tailed. The diagnostic cut off value for parameters (BNP, left ventricular end-systolic diameter, left end-diastolic diameter, left ventricular end-diastolic volume, left ventricular end-systolic volume, cycle length of induced VT, cycle length of induced VF) was calculated by means of receiver-operating characteristics (ROC) curves. The values of EPS were analysed with ROC curve, chi-square or Fisher's exact test for prediction of appropriate ICD therapy. The Kaplan-Meier analysis with a log-rank test was used to compare the probability of ICD therapy. A multivariate Cox regression analysis was performed on variables regarded as significant predictors (p < 0.1) with a univariate analysis for appropriate an ICD therapy. A P value of ≤0.05 was considered as significant. The statistical package used was SPSS 12.0 for Windows.

## Results

During a mean follow-up of 32.9 ± 16.1 months an appropriate ICD therapy occurred in 43 (46%) patients and an inappropriate ICD therapy occurred in 9 (10 %) patients. The clinical characteristics of the three groups were similar (Table 1). Patients of group II had a higher incidence of a posterior wall infarction and a lower incidence of an anterior wall infarction than those of the other groups (Table 1). The echocardiographic parameters were also similar with the exception of a larger left ventricular end-diastolic and end-systolic diameter of group I than of group II (Table 2).

### Incidence of ICD therapy

**Figure 1:**
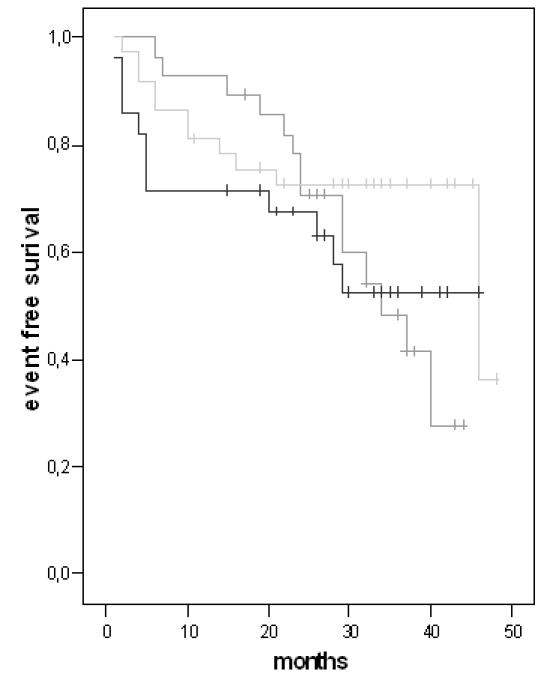
Kaplan Meier Analysis for appropriate ICD therapy. (group I = light-grey line, group II = black line, group III = dark-grey line) long-rank P = 0.24 for group I vs. II, long-rank P = 0.17 for group I vs. III, long-rank P = 0.33 for group II vs. III.

The incidence of an appropriate (group I 40%, group II 54% and group III 48% of patients) and inappropriate (group I 10 %, group II 8%, group III 11% of patients) ICD therapy was similar between the three groups. The long-rank test was also similar between the three groups (Figure 1). The number of treated VT's (group I 25%, group II 30% and group III 28% of patients) or VF (group I 15%, group II 24% and group III 20% of patients) did also not differ.

### Comparison of patients with and without ICD therapy

Patients with an appropriate ICD therapy had a significantly higher BNP value, a higher incidence of a sotalol, digitalis and ACE inhibitor/angiotensin receptor antagonists therapy than patients without an appropriate ICD therapy (Table 3). The echocardiographic parameters showed a significant left ventricle enlargement in patients with an appropriate ICD therapy and a similar left ventricular ejection fraction between patients with and without an ICD therapy (Table 4).

**Figure 2:**
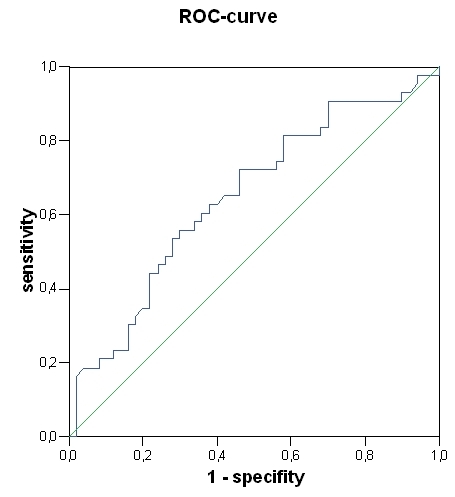
Receiver-operating characteristics curves of BNP (area under the curve 0.647, P = 0.015)

**Figure 3:**
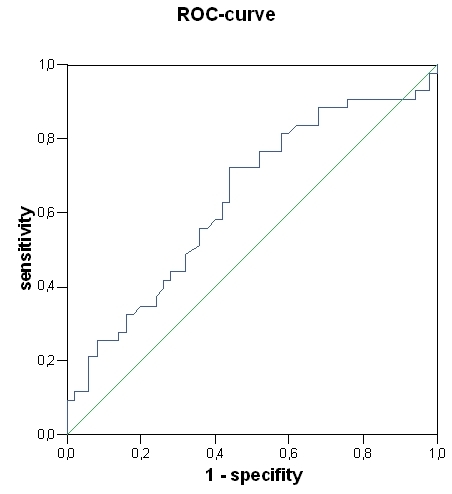
Receiver-operating characteristics curves of left ventricular end-diastolic volume (area under the curve 0.637, P = 0.023)

**Figure 4:**
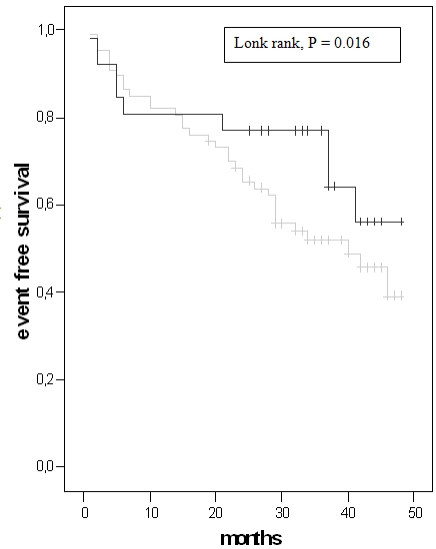
Kaplan Meier analysis for BNP (BNP ≥ 265 pg/ml = black line, BNP < 265 pg/ml = grey line)

The ROC curve analysis revealed a threshold for left ventricular end-diastolic volume (172 ml, AUC 0.637, P = 0.023) and BNP (265 pg/ml, AUC 0.647, P = 0.015) for a significant prediction of appropriate ICD therapy (Figure 2, 3). Left ventricular end-systolic diameter, left end-diastolic diameter, left ventricular end-systolic volume, cycle length of induced VT and cycle length of induced VF did not achieve a significant value. The long-rank test showed a significant difference for BNP ≥ 265 pg/ml (P = 0.016) (Figure 4). The incidence of appropriate ICD therapies was significantly higher in patients with a BNP ≥ 265 pg/ml compared to in patients with a BNP < 265 pg/ml [27 vs. 16 patients (60 vs. 33%), P = 0.013]. BNP (265 pg/ml) predicted an appropriate ICD therapy with a 3.5-fold risk (P = 0.017).

**Figure 5:**
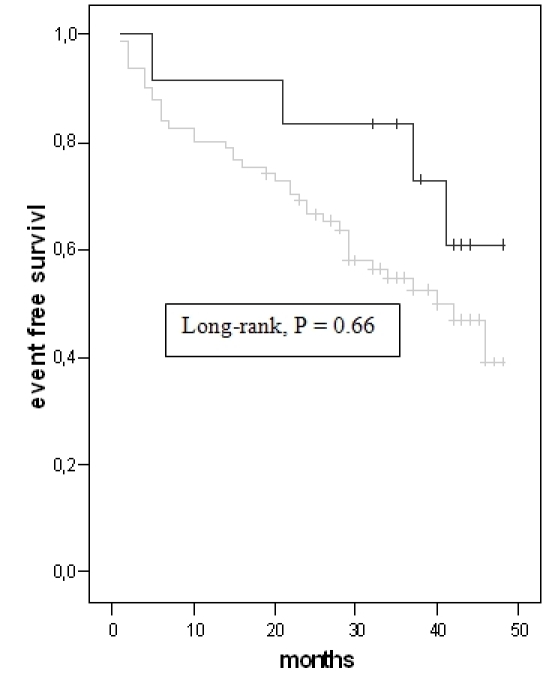
Kaplan Meier analysis for left ventricular end-diastolic volume (LVEDV ≥ 172 ml = black line, LVEDV < 172 ml = grey line)

The Kaplan-Meier analysis did not achieve a significant difference for left ventricular end-diastolic volume ≥ 172 ml (P = 0.66) (Figure 5).

**Figure 6:**
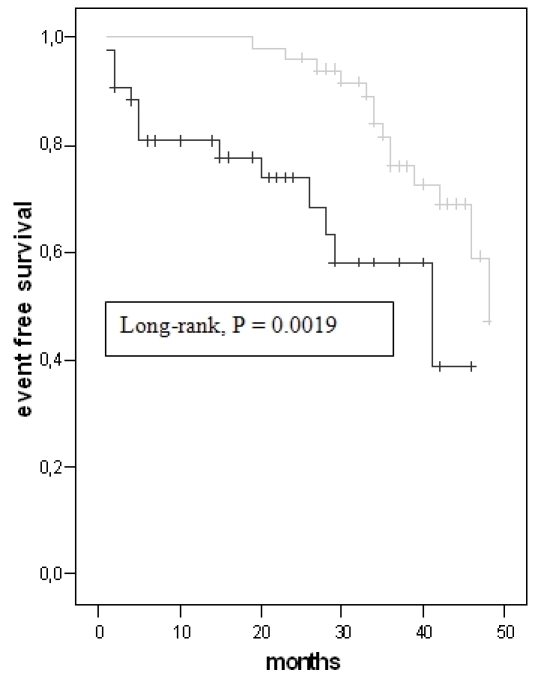
Kaplan Meier analysis for abnormal EPS (abnormal EPS = black line, normal EPS = grey line)

The prediction of an appropriate ICD therapy was achieved by an abnormal EPS. Patients who fulfilled the definition had a significantly higher incidence of an appropriate ICD therapy than patients who did not fulfil the definition (85 vs. 27% of patients, P = 0.005). The results of  group II with abnormal EPS results could not be assigned to group III patients. 13 patients fulfilled the definition of abnormal EPS but only 5 received an ICD therapy.  An abnormal EPS did not achieve a significant difference between patients with and without ICD therapy (38 vs. 50% of patients). The Kaplan-Meier analysis showed a significant difference between patients with normal and abnormal EPS (Figure 6). EPS values of non-inducible patients could not predict an appropriate ICD therapy.

### Prediction of death

There was no significant difference between dead and alive patients in clinical characteristics with the exception of a higher BNP level in dead than alive patients (421.5 ± 433.4 vs. 307.5 ± 188.3 pg/ml, P = 0.011). A prediction of death could not be achieved by any parameter.

## Discussion

In the present retrospective study we showed that MADIT II like patients did not differ in occurrence of an appropriate ICD therapy in relation to a performance of an EPS. Only patients with an induction of ventricular arrhythmias benefited from an EPS for a prediction of an appropriate ICD therapy. BNP was significantly higher in patients with an appropriate ICD and was able to predict an appropriate ICD therapy. Patients with an appropriate ICD therapy showed a left ventricular enlargement without the possibility to predict an appropriate ICD therapy.

### Electrophysiological study and appropriate ICD therapy

An EPS was not suitable to predict an appropriate ICD therapy in patients with who underwent an EPS. A prediction of an appropriate ICD therapy was only achieved in a subgroup of patients with inducibility of ventricular arrhythmias in EPS. Patients without an induction of a ventricular arrhythmia also had a high incidence of ventricular arrhythmias in our study that was also found in similar studies [7,10-18]. A subgroup analysis of MADIT II like patients who underwent an EPS showed that inducible and non-inducible patients had similar ICD therapies (long-rank P = 0.280) [7]. Furthermore inducible patients had a greater likelihood of experiencing ICD therapy for VT than non-inducible patients and ICD therapy for VF was more common in non-inducible patients than in inducible patients [7]. Similar results were also observed in Antiarrhythmics versus Implantable Defibrillators (AVID) trial [11]. In the AVID trial patients with induction of a slow VT (< 200 bpm) showed a significantly higher incidence of appropriate ICD therapy (P = 0.004) [11]. The results of the Cardiac Arrest Study Hamburg (CASH) indicated that patients with an EF < 35% did not differ in all-cause death or sudden cardiac death in comparison to patients with inducible or non-inducible in EPS [17]. A prediction of all-cause death and sudden cardiac death by EPS was achieved in patients with an EF > 35% [18]. Ruppel et al. observed a weak prediction of EPS for recurrence of VF and VT in patients who survived an episode of VF [10].

In contrast to the results of the previous studies other authors found a prediction of appropriate ICD therapy by induction of VT or VF with EPS [10,12-16,18] but without a definition of an abnormal EPS constellation. The most meaningful example for the prediction of an appropriate ICD therapy by EPS was the Multicenter Unsustained Tachycardia Trail (MUSTT), which showed a lower risk of appropriate ICD therapy in patients without induction in EPS [13]. But it was remarkable that non-inducible patients had a high incidence of appropriate ICD therapy in studies in which EPS could well predict an appropriate ICD therapy [10,12-14,16-18].

Only patients who fulfilled the definition of an abnormal EPS reached a significantly higher incidence of appropriate ICD therapy than patients who did not fulfil the definition in our study. To the best of our knowledge this was the first study with a prediction of an appropriate ICD therapy by values of EPS. However, an EPS could predict an appropriate ICD therapy and particularly in regard to an abnormal EPS. But non-inducible patients also had a high risk for an appropriate ICD therapy.

### Brain natriuretic peptide

BNP was significantly higher in patients with appropriate ICD therapy and reached a prediction of a BNP appropriate ICD therapy in MADIT II like patients. A prediction of death could not be achieved by our study. However, BNP was an excellent predictor for sudden death and heart failure [19-23]. In addition, Verma et al. also observed a higher value of BNP in patients with an appropriate ICD therapy (573 vs. 243 pg/ml, P = 0.0003) and predicted an appropriate ICD therapy by a BNP value of 263 pg/ml (long rank P = 0.006) in their study [24]. Manios et al. found a higher NT-proBNP value in patients with an EF < 35% (997.27 vs. 654.87 pmol/l, P = 0.001) and predicted an appropriate ICD therapy with a NT-proBNP value of 880 pmol/l with a sensitivity of 73%, a specificity of 88%, a positive predictive value of 80% and a negative predictive value of 88% [25]. In consensus of these studies BNP was an excellent predictor for sudden death, heart failure and appropriate ICD therapies.

### Limitations

The number of patients is lower in comparison to the larger trials like MADIT II, AVID or CASH in our study. The evaluation of the EPS parameters and BNP value were performed retrospectively. Our preliminary observations are important aspects for a prospective trial of the predictive power of abnormal EPS and BNP value. The three groups were comparable in their clinical characteristics except for the location of myocardial infarction. To the best of our knowledge the location of myocardial infarction did not influence the outcome of patients with ICD's.

In conclusion, our study results suggest that abnormal EPS and BNP value predicted an appropriate ICD therapy in MADIT II like patients. The abnormal EPS could only be applied in patients with an induction of ventricular arrhythmia in EPS. Many patients who received an appropriate ICD therapy were non-inducible in EPS or got an ICD implanted without an EPS according to the MADIT II criteria. Thus MADIT II patients were high-risk patients for an appropriate ICD therapy and should be implanted an ICD independent from an EPS.

## Figures and Tables

**Table 1:**
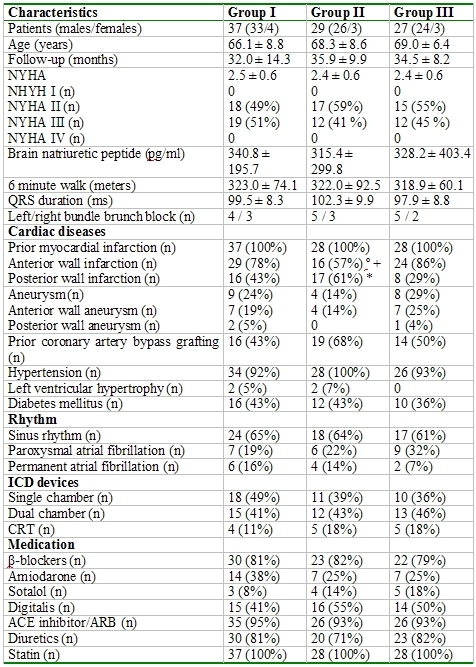
Patients characteristics in comparison to electrophysical study

**Table 2:**
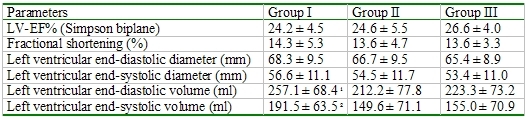
Comparison of echocardiographic parameters to electrophysical study

**Table 3:**
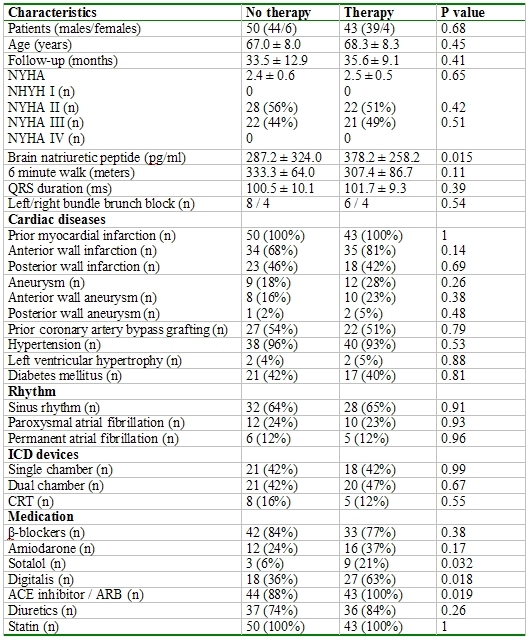
Patients characteristics in comparison to appropriate ICD therapy

**Table 4:**
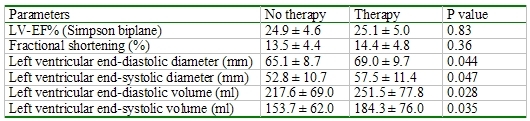
Comparison of echocardiographic parameters to appropriate ICD therapy
